# Disruption of white matter integrity and its relationship with cognitive function in non-severe traumatic brain injury

**DOI:** 10.3389/fneur.2022.1011304

**Published:** 2022-10-11

**Authors:** Aimi Nadhiah Abdullah, Asma Hayati Ahmad, Rahimah Zakaria, Sofina Tamam, Aini Ismafairus Abd Hamid, Wen Jia Chai, Hazim Omar, Muhammad Riddha Abdul Rahman, Diana Noma Fitzrol, Zamzuri Idris, Abdul Rahman Izaini Ghani, Wan Nor Azlen Wan Mohamad, Faiz Mustafar, Muhammad Hafiz Hanafi, Mohamed Faruque Reza, Hafidah Umar, Mohd Faizal Mohd Zulkifly, Song Yee Ang, Zaitun Zakaria, Kamarul Imran Musa, Azizah Othman, Zunaina Embong, Nur Asma Sapiai, Regunath Kandasamy, Haidi Ibrahim, Mohd Zaid Abdullah, Kannapha Amaruchkul, Pedro Antonio Valdes-Sosa, Maria Luisa Bringas Vega, Bharat Biswal, Jitkomut Songsiri, Hamwira Sakti Yaacob, Putra Sumari, Nor Azila Noh, Azlinda Azman, Paramjit Singh Jamir Singh, Jafri Malin Abdullah

**Affiliations:** ^1^Department of Physiology, School of Medical Sciences, Universiti Sains Malaysia, Kota Bharu, Malaysia; ^2^Brain and Behaviour Cluster, School of Medical Sciences, Universiti Sains Malaysia, Kota Bharu, Malaysia; ^3^Faculty of Science and Technology, Universiti Sains Islam Malaysia, Nilai, Malaysia; ^4^Department of Neurosciences, School of Medical Sciences, Universiti Sains Malaysia, Kota Bharu, Malaysia; ^5^Hospital Universiti Sains Malaysia, Universiti Sains Malaysia, Kota Bharu, Malaysia; ^6^Department of Community Medicine, School of Medical Sciences, Universiti Sains Malaysia, Kota Bharu, Malaysia; ^7^Department of Pediatrics, School of Medical Sciences, Universiti Sains Malaysia, Kota Bharu, Malaysia; ^8^Department of Ophthalmology, School of Medical Sciences, Universiti Sains Malaysia, Kota Bharu, Malaysia; ^9^Department of Radiology, School of Medical Sciences, Universiti Sains Malaysia, Kota Bharu, Malaysia; ^10^Gleneagles Hospital Kuala Lumpur, Kuala Lumpur, Malaysia; ^11^School of Electrical and Electronic Engineering, Universiti Sains Malaysia, Nibong Tebal, Malaysia; ^12^Graduate School of Applied Statistics, National Institute of Development Administration (NIDA), Bangkok, Thailand; ^13^The Clinical Hospital of Chengdu Brain Science Institute, MOE Key Lab for Neuroinformation, University of Electronic Science and Technology of China, Chengdu, China; ^14^The Cuban Neurosciences Center, La Habana, Cuba; ^15^Department of Biomedical Engineering, New Jersey Institute of Technology, Newark, NJ, United States; ^16^EE410 Control Systems Laboratory, Department of Electrical Engineering, Faculty of Engineering, Chulalongkorn University, Bangkok, Thailand; ^17^Department of Computer Science, Kulliyah of Information and Communication Technology, International Islamic University Malaysia, Kuala Lumpur, Malaysia; ^18^School of Computer Sciences, Universiti Sains Malaysia, Penang, Malaysia; ^19^Faculty of Medicine and Health Sciences, Universiti Sains Islam Malaysia, Nilai, Malaysia; ^20^School of Social Sciences, Universiti Sains Malaysia, Penang, Malaysia

**Keywords:** traumatic brain injury, diffusion MRI, fractional anisotropy, neuropsychological test, tract-based spatial statistic

## Abstract

**Background:**

Impairment in cognitive function is a recognized outcome of traumatic brain injury (TBI). However, the degree of impairment has variable relationship with TBI severity and time post injury. The underlying pathology is often due to diffuse axonal injury that has been found even in mild TBI. In this study, we examine the state of white matter putative connectivity in patients with non-severe TBI in the subacute phase, i.e., within 10 weeks of injury and determine its relationship with neuropsychological scores.

**Methods:**

We conducted a case-control prospective study involving 11 male adult patients with non-severe TBI and an age-matched control group of 11 adult male volunteers. Diffusion MRI scanning and neuropsychological tests were administered within 10 weeks post injury. The difference in fractional anisotropy (FA) values between the patient and control groups was examined using tract-based spatial statistics. The FA values that were significantly different between patients and controls were then correlated with neuropsychological tests in the patient group.

**Results:**

Several clusters with peak voxels of significant FA reductions (*p* < 0.05) in the white matter skeleton were seen in patients compared to the control group. These clusters were located in the superior fronto-occipital fasciculus, superior longitudinal fasciculus, uncinate fasciculus, and cingulum, as well as white matter fibers in the area of genu of corpus callosum, anterior corona radiata, superior corona radiata, anterior thalamic radiation and part of inferior frontal gyrus. Mean global FA magnitude correlated significantly with MAVLT immediate recall scores while matrix reasoning scores correlated positively with FA values in the area of right superior fronto-occipital fasciculus and left anterior corona radiata.

**Conclusion:**

The non-severe TBI patients had abnormally reduced FA values in multiple regions compared to controls that correlated with several measures of executive function during the sub-acute phase of TBI.

## Introduction

Traumatic brain injury (TBI) poses an increased risk for early decline in neurological function, either temporarily or, in worse situations, could result in permanent incapacitation (1–3), making it a vital public health concern (4–6). TBI is often classified as mild, moderate or severe according to injury severity (7). Moderate TBI is often collectively classified with severe level of brain injury, categorizing it as a hybrid moderate-to-severe TBI (7) or in some cases, with the mild type of brain injury, mild-to-moderate TBI (8). In terms of timing post injury, the categories include acute (within 1 week), subacute (1 week to 3 months), and chronic (more than 3 months) (9). However, the grading of TBI often does not reflect the severity of the actual injury and the subsequent impact on cognitive function (10). The majority of current categories are founded on clinical evaluations that are often poor predictors of long-term disability (11). A novel strategy based on multimodal quantifiable data (such as imaging and biomarkers) and risk-labels would be advantageous for patients as well as clinical TBI research (11).

Regardless of the severity of brain injury, TBI is often accompanied by diffuse axonal injury, a form of shearing injury in which the white matter of the brain is damaged (12, 13). However, due to its microscopic size, the resulting diffuse axonal injury is hardly detectable on computed tomography (CT) and conventional magnetic resonance imaging (MRI). Diffusion MRI has been proposed as the best technique to detect microstructural changes to white matter, enabling diffuse axonal injury to be more easily identified (14, 15).

Diffusion MRI is an indirect measure of white matter integrity obtained by quantifying changes in the diffusivity of water molecules within fiber tracts (16). Theoretically, in a healthy brain, water molecules preferentially move parallel to axonal fibers where the diffusion is constrained and restricted. This condition is referred to as anisotropic diffusion (14) and is indicated by higher fractional anisotropy (FA) values. On the other hand, in the injured brain, where fiber tracts are disrupted, water molecules tend to move more freely in all directions making the diffusion relatively unconstrained, thus referred to as isotropic diffusion (17, 18) and indicated by lower FA values. Computed from diffusion MRI, the FA index represents fiber tract integrity through the relative differences of diffusion along and across axonal fibers, where reduction in FA values corresponds with a local loss of structural white matter integrity (19). Thus, recent studies used FA as a relevant biomarker to detect diffuse axonal injury, which may have prognostic value in TBI (20, 21). Another parameter measured in diffusion MRI is mean diffusivity (MD), which is the average of the diffusion measurements along the three axes (7).

Diffuse axonal injury, a result of acceleration and deceleration of the brain inside the hard skull causing shearing of the long axonal fibers, is frequently found in all types of brain injury and comparatively examined together with cognitive performance; whether in mild TBI (22–25) or severe TBI (26). A study on moderate-severe TBI patients performed five decades after injury using diffusion MRI and voxel-based morphometry revealed a persistence of brain microstructural alterations with late cognitive sequelae (27). A metaanalysis of 20 studies concluded that while diffusion MRI parameters are associated with cognitive performance, most findings were based on single studies and in need of replication (28). Therefore, the main aim of this study was to add to this body of knowledge in investigating the level of impairment of white matter integrity in patients with non-severe TBI in the subacute phase. This study also aimed to analyze the correlation between FA values and neuropsychological scores in this group of patients. The findings of this research could aid in the development of new diagnostic criteria for TBI patients.

## Materials and methods

### Patients

This study involved 11 male patients (mean age, 27.5 ± 13.2, range 18–53 years) with non-severe, comprising mild and moderate TBI recruited from the Emergency Department, Hospital Universiti Sains Malaysia (HUSM). The inclusion criteria were: (i) age between 18 and 65 years old, (ii) 9 years education and above, (iii) right-hand dominant assessed using the Edinburgh Handedness Inventory (29, 30), (iv) no psychiatric illness and not consuming any psychiatric drugs, (v) brain damage caused by brain injury (not due to a surgical procedure) which is blow/ripping involving left and right side of fronto-temporal-parietal lobes as first diagnosed by computed tomography scan, and Glasgow Coma Scale (GCS) of 8–12 (moderate TBI) and 13–15 (mild TBI), whereas exclusion criteria were: (i) under treatment with any medications that may compromise working memory process, (ii) presence of injuries to eyes and ears, and (iii) have cracked skull, scalp injury or fractures. The etiologies of the TBI included road traffic accident, fall, and blunt force. Age-matched control volunteers (11 volunteers; all male; mean age 28.4 ± 10.2, range 20–52 years) were recruited from advertisements placed around the university, social media websites, and from word of mouth (Table 1). Inclusion criteria for healthy participants were similar except for the absence of TBI. The study was approved by the Human Research Ethics Committee of Universiti Sains Malaysia (USMJEPeM/15110486), and all subjects provided written informed consent.

**Table 1:** Demographic results of TBI and healthy controls.

	**Patients** ***n* = 11**	**Controls** ***n* = 11**
Age (years)	27.5 ± 13.2	28.4 ± 10.2
Education (years)	13.6 ± 3.0	15.2 ± 2.2
Time post injury (days)	41.3 ± 8.9	

### Study design and study procedures

This is a case-control prospective study. TBI patients had CT scans performed immediately following injury. Diffusion MRI scanning was performed within 10 weeks post injury. Neuropsychological assessment was performed during the MRI scanning appointment in both patients and age-matched controls.

### Neuropsychological assessment

Several tests were administered to assess executive function, verbal and visual memory, visuoperception and processing speed, all common deficits following TBI. The assessment was held within 10 weeks of injury for moderate TBI patients and included the Wechsler Abbreviation Scale of Intelligence (WASI) block design for motor skill and matrix reasoning test for visuo-spatial problem solving (31), the validated Malay version of the Auditory Verbal Learning Test (MAVLT) for verbal memory (32), the Wisconsin Card Sorting Test (WCST) to measure problem-solving and perseverative responding (33), the Rey Complex Figure Test (RCFT) to assess visuospatial or constructional ability and visual memory (34), and the Comprehensive Trail Making Test (CTMT) for visuomotor speed and maintenance of cognitive set-shifting (35).

### Data acquisition and imaging parameters

Whole-brain conventional and diffusion imaging were acquired on a 3.0 T Philips Achieva MRI scanner (Netherlands), available in the Department of Radiology, HUSM with a 32-channel SENSE head coil for pulse transmissions and signal reception. T1-weighted images were obtained *via* conventional MR imaging with the following protocol (TR = 7.4 ms, TE = 3.4 ms, FOV = 250 × 250, matrix size = 228 × 227). Diffusion-weighted images were sensitized with a b-value of 1,000 s/mm^2^, using echo-planar (EPI) sequence (TR = 10,726 ms, TE = 76 ms, slice thickness = 2.3 mm, FOV = 230 × 230 mm, matrix size = 96 × 94).

### Data pre-processing

Data preprocessing and analysis were performed using FMRIB's Software Library [FSL; (36)] version 5.0.9; Oxford Center for Functional MRI of the Brain (FMRIB), UK; http://www.fmrib.ox.ac.uk/fsl/. Diffusion images were initially registered to the *b* = 0 image by affine transformation to minimize the distortion caused by the effect of motion and eddy current in the gradient coils using the eddy current correction function in FMRIB's Diffusion Toolbox v3.0 (FDT). The registered images were skull-stripped using the Brain Extraction Tool [BET (37)] and FA images generated using the FDT (38).

After the removal of skull and non-brain tissues, next step were involved calculating diffusion tensor by fitting the tensor model at each voxel using a simple least square fit to the diffusion data (39). This step produced 10 outputs include of tensor eigenvalues (that when calculated represented diffusion magnitude/strength in the primary, secondary and tertiary diffusion directions), eigenvectors (described diffusion orientation represented by principal and radial directions), mean diffusivity (MD) (molecular diffusion rate), fractional anisotropy (FA) map (the variance of the 3 eigenvalues/magnitude normalized which measure the strength of directional preference), mode of the anisotropy (MO) images as well as an image of raw T2 signal with no diffusion weighting (SO) (40). This step was achieved through DTIFIT.

Upon completing DTIFIT step for each participant diffusion data, the output of FA map from patient and control participant were selected and copied into a new-empty directory.

#### Tract-based spatial statistics (TBSS)

Voxelwise statistical analysis of the diffusion-weighted data was carried out using tract-based spatial statistics [TBSS; (39)], part of FSL. The approach is to align multiple FA images using voxelwise non-linear registration with intermediate degrees of freedom, and then projecting the data onto a tract skeleton (39).

After fractional anisotropy maps calculation for each subject, voxel-wise statistical analysis was carried out through TBSS. All FA images underwent non-linear registration by aligning them to an FMRIB58_FA 1 × 1 × 1 mm standard space image. This step involved one registration being carried out per subject and commonly gives good alignment results. Next, the standard space FA images were merged into a single 4D image to create a mean FA image so that it can be fed into the FA skeletonisation step. This would generate a mean FA skeleton that represents the center of all tracts common to the entire group (2). The mean FA skeleton map was visually inspected. A good registration result could be identified by the majority of the tracts of each subject being reasonably well-aligned to the relevant parts of the skeleton. Next, this was thresholded to a standard value of 0.2 to include major white matter pathways but to exclude enough peripheral tracts where there was significant inter-subject variability and partial volume effects with gray matter (2). The aligned FA image for each subject was then projected onto the resulted binary skeleton mask by filling the skeleton with FA values from the closest relevant tract center. The resulting skeletonised data was then fed into voxelwise cross-subject statistics.

### Statistical analysis

Group comparison was performed using Randomize tool v2.9 from FSL. The Threshold-Free Cluster Enhancement (TFCE) option was used to define the clusters (39). The TFCE method is technically more robust than cluster-based thresholding as well as it does not necessarily opt to decide arbitrary initial cluster-forming threshold. Monte Carlo permutation testing was performed where it generated *n* = 1,000 random permutations. The statistical threshold was set at *p* < 0.05 FamilyWise Error (FWE) corrected. Areas corresponding to significant clusters were identified using the JHU White-Matter Tractography atlas. Mean FA values were obtained from the skeleton map of each subject.

Statistical test on non-imaging data were performed using SPSS version 26.0 (Statistical Package for the Social Sciences) (IBM Corp., Armonk, NY, United States). The Shapiro-Wilk W-test was used to test for normality distribution of all continuous variables. Group differences for IQ, block design and matrix reasoning (subtests of WASI-II), MAVLT, WCST and RCFT delayed recall scores were examined using the independent two-sample *t*-test since the data were normally distributed using a significance level of *p* < 0.05, and for non-normal distribution of RCFT immediate recall and CTMT scores as well as age and education level were examined using the Mann-Whitney U test. The relationship between imaging metrics and neuropsychological measures was explored using Pearson's correlation.

## Results

### Demographic and neuropsychological test results of TBI and healthy controls

Three out of the 11 TBI patients sustained mild TBI while the rest had moderate TBI. Table 1 shows the demographic characteristics of TBI and healthy control groups while Table 2 shows the site and type of lesion of TBI patients. The mean age and education level were not significantly different between the two groups.

**Table 2:** Site and type of lesion in TBI patients during initial CT scan.

**Patient**	**Age**	**Affected hemisphere**	**Lesion**
1	53	Left	Subarachnoid hemorrhage around left frontal and parietal lobes. No brain parenchyma injury
2	19	Left	Left EDH
3	18	Left	Left frontal contusion, left small EDH
4	18	Left	Left frontal contusion, small right temporal contusion
5	19	Left	Left thin EDH, left temporal contusion (bilateral temporal contusions)
6	22	Left	Left temporal base EDH
7	53	Left	Left parietal contusion, traumatic SAH
8	19	Right	Right thin frontal EDH
9	29	Left	Left temporal EDH
10	25	Right	Right frontal contusion
11	27	Left	Left convexity acute SDH

SAH, subarachnoid hemorrhage; EDH, extradural hemorrhage; SDH, subdural hemorrhage.

### Neuropsychological performances between TBI patients and healthy controls

Neuropsychological performance between TBI and healthy groups showed that several assessments reached statistically significant difference between the two groups (Table 3), which included WASI's IQ and Matrix Reasoning tests, MAVLT immediate recall, and both RCFT immediate and delayed recall. CTMT almost reached significant difference statistically (*p* = 0.056) while other tests showed no significant performance difference between the groups.

**Table 3:** Neuropsychological performance for TBI and control groups.

	**TBI group**		**Control group**		***p*-value**
	**Mean**	**SD**	**Mean**	**SD**	
IQ	93.4	11.7	103.6	9.2	**0.033**
Block design	49.1	6.4	51.0	9.7	0.591
Matrix Reasoning	42.5	12.7	53.8	4.5	**0.016**
MAVLT_immediate recall	42.8	11.5	53.1	6.1	**0.019**
MAVLT_delayed recall	8.9	3.9	11.4	2.1	0.083
WCST	84.7	19.0	88.4	26.6	0.716
RCFT_immediate recall	40.01	20.2	59.7	6.5	**0.040**
RCFT_delayed recall	38.1	17.4	54.0	9.4	**0.017**
CTMT	33.8	17.5	43.3	9.1	0.056

MAVLT, malay-version of auditory verbal learning test; WCST, wisconsin card sorting test; RCFT, rey complex figure test; CTMT, comprehensive trail making test. Significant values for comparison between healthy subjects and patient are in bold.

### FA differences between TBI and healthy control groups

**Figure 1:**
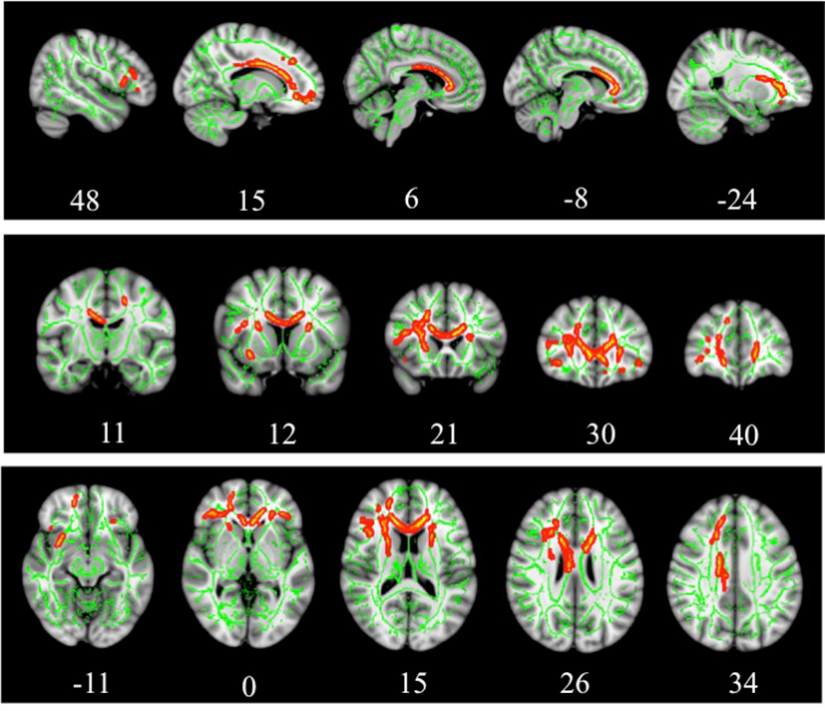
TBSS analysis of white matter skeleton. Voxels demonstrating significantly (*p* < 0.05) decreased FA values for the subjects with TBI compared with the control group are shown in red-yellow. Voxels were thickened into local tracts and overlaid on the white matter skeleton (green).

Results of whole-brained TBSS comparative analysis of DTI between 11 TBI and 11 healthy controls revealed multiple areas of significant FA reductions in TBI patients as compared to control (Figure 1). A few long association fibers were affected including superior fronto-occipital fasciculus, superior longitudinal fasciculus, uncinate fasciculus, and cingulum, as well as white matter fibers in the area of genu of corpus callosum, anterior corona radiata, superior corona radiata, anterior thalamic radiation and part of inferior frontal gyrus. Detailed information for each cluster, the anatomic location, voxel coordinates and total number of voxels is presented in Table 4. There were no significant areas where FA values were increased in patients compared to controls.

**Table 4:** Anatomic location of decreased FA clusters in the TBI groups compared to controls.

**Hemisphere**	**Anatomic location**	**Voxel coordinates**			***p*-value**	**Cluster size**
		**X**	**Y**	**Z**		
	Genu of corpus callosum	98	153	85	0.034	2,324
Right	Superior fronto-occipital fasciculus	67	125	94	0.035	2,090
Left	Anterior corona radiata	114	156	79	0.046	474
Right	Superior longitudinal fasciculus	41	144	77	0.048	432
Right	Uncinate fasciculus	59	136	61	0.046	148
Right	Anterior thalamic radiation	59	166	89	0.049	88
Left	Superior corona radiata	107	115	117	0.045	58
Right	Cingulum	78	94	103	0.05	49
Left	Inferior frontal gyrus	115	157	59	0.05	16
	Genu of corpus callosum	101	151	71	0.049	14

**Figure 2:**
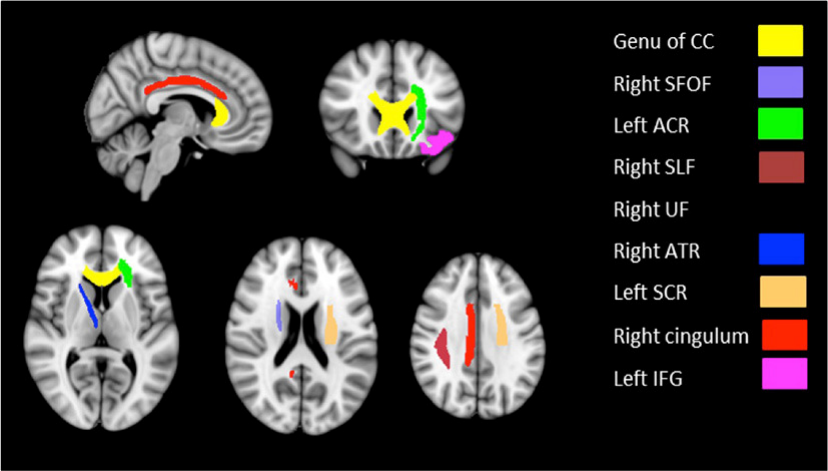
Masks of nine ROIs selected from the results of whole-brain group comparison overlaid on MNI152 template brain.

Based on the whole-brain group comparison of FA values, we selected 9 areas in the brain as our regions of interest (ROI). Figure 2 illustrates the masks of ROIs. We obtained the mean FA values of the whole-skeletonized brain and all the selected ROIs. Whole-brain and several ROIs reached statistical significance with *p* < 0.005 (Table 5).

**Table 5:** Group differences in mean FA from the whole skeletonised brain and the ROIs.

	**TBI group**		**Control group**		***p*-value**
	**Mean**	**SD**	**Mean**	**SD**	
FA global	0.579	0.021	0.623	0.015	**0.000**
FA genu of CC	0.180	0.004	0.185	0.001	**0.000**
FA right SFOF	0.077	0.004	0.083	0.002	**0.000**
FA left ACR	0.175	0.008	0.182	0.006	**0.017**
FA right SLF	0.177	0.007	0.178	0.006	0.767
FA right UF	0.125	0.008	0.133	0.005	**0.020**
FA right ATR	0.186	0.008	0.190	0.008	0.184
FA left SCR	0.158	0.005	0.160	0.006	0.576
FA right cingulum	0.093	0.004	0.096	0.004	0.142
FA left IFG	0.047	0.003	0.051	0.005	0.076

Significant values for comparison between healthy subjects and patient are in bold.

### Correlation between FA values and neuropsychological assessments in TBI patients

Correlation analysis was performed between the FA values within the ROIs with the neuropsychological scores that were significantly different between the TBI and control groups while controlling for age and years of education factors. Results showed that the mean global FA measure correlated significantly with MAVLT immediate recall scores (*r* = 0.675, *p* = 0.001). The correlation of global FA maps with other psychological test scores did not reach statistical significance.

**Figure 3:**
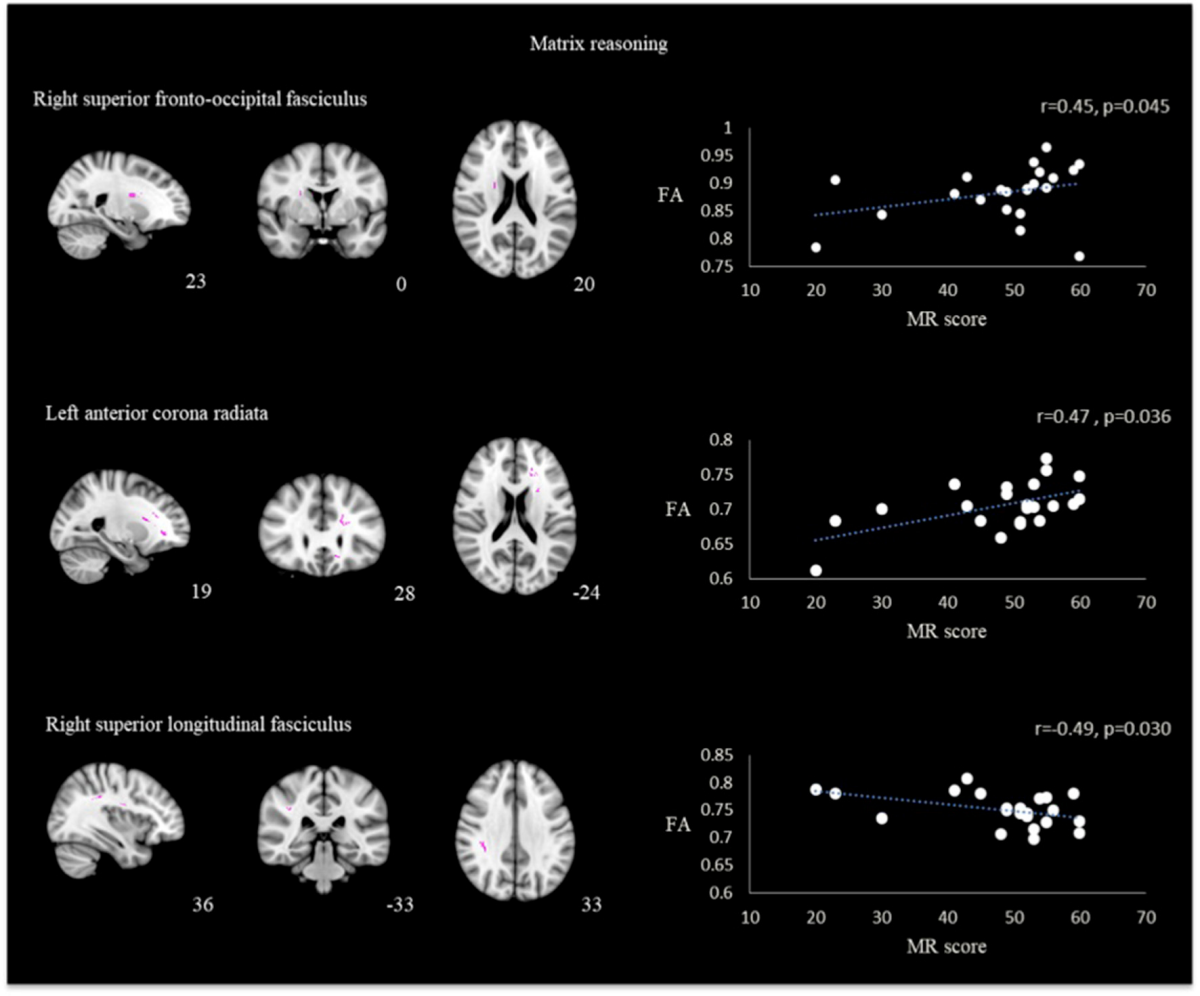
Correlation between FA and matrix reasoning scores in the region of right superior fronto-occipital fasciculus, left anterior corona radiata and right superior longitudinal fasciculus in TBI patients.

For the correlation using the ROIs, matrix reasoning scores correlated positively with FA values in the area of right superior fronto-occipital fasciculus (*r* = 0.45, *p* = 0.045) and left anterior corona radiata (*r* = 0.47, *p* = 0.036) while having negative correlation in the area of right superior longitudinal fasciculus (*r* = −0.49, *p* = 0.030) (Figure 3).

**Figure 4:**
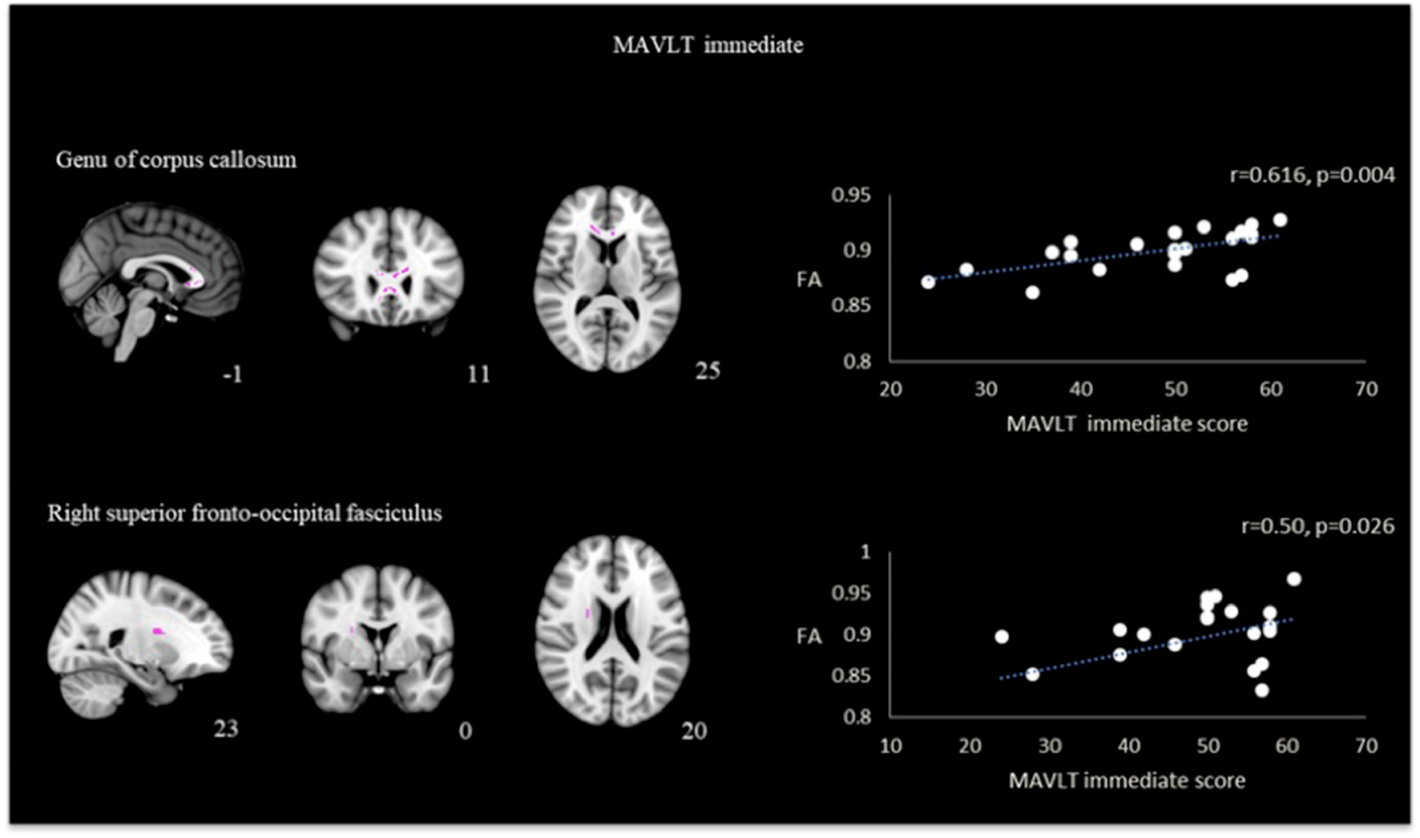
Correlation between FA and MAVLT immediate recall in the region of genu of corpus callosum and right superior fronto-occipital fasciculus in TBI patients.

A positive correlation was also found between memory performance assessed by MAVLT immediate recall with the FA skeletonized genu of corpus callosum (*r* = 0.62, *p* = 0.004) and right superior fronto-occipital fasciculus (*r* = 0.50, *p* = 0.026). Results presented in Figure 4. No other correlation reached statistical significance in both groups.

### Correlation between MD values and neuropsychological assessments in TBI patients

**Figure 5:**
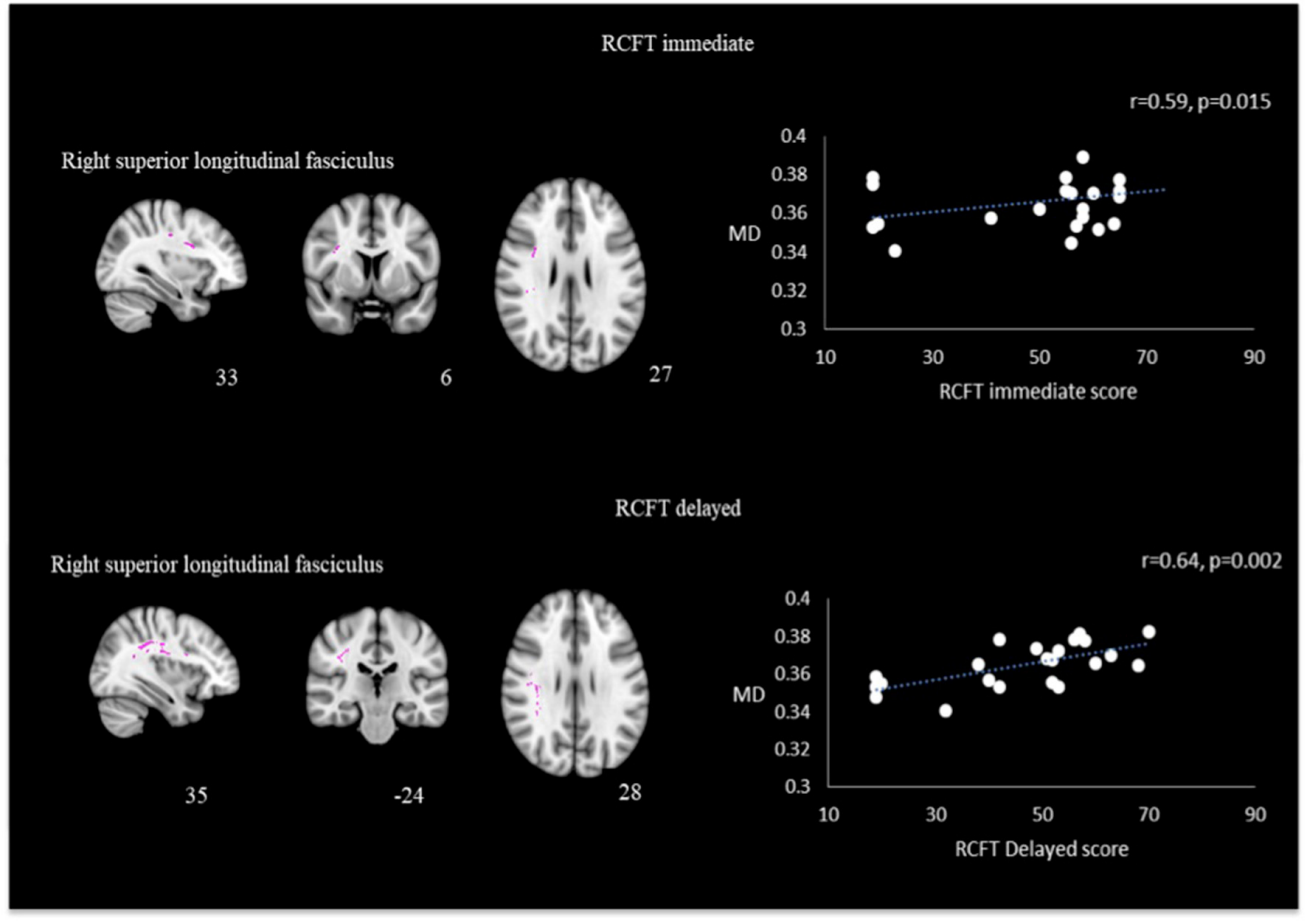
Correlation between MD and RCFT immediate and delayed scores in the right superior longitudinal fasciculus in TBI patients.

Utilizing identical ROIs as per FA analysis, correlation analysis performed between MD values with psychological measurement revealed that the right superior longitudinal fasciculus had significant positive correlation with RCFT immediate recall (*r* = 0.593, *p* = 0.015) and RCFT delayed recall (*r* = 0.640, *p* = 0.002). Results are presented in Figure 5. Other correlation did not reach statistical significance.

## Discussion

In this study, we used diffusion MRI to assess the level of impairment of structural white matter integrity in the brain of non-severe, mild-moderate TBI. Although primary CT scan findings showed parenchymal injury of the left side of the brain in the majority of the TBI patients, TBSS analysis revealed that white matter disruptions extended across to the right hemisphere. Whole-brain diffusion analysis revealed a global decrease in mean FA values in patients with the involvement of several white matter tracts. Anatomical peak voxels based on cluster analysis showed that the FA reductions were on both sides of the brain. While CT scan is the mainstay of imaging for initial assessment of acute TBI (41) and is of value in assessing bones as well as detecting acute subarachnoid or acute parenchymal hemorrhage, it is inferior in detecting potential cerebral pathology in TBI patients (42). A study on TBI patients found that out of 450 patients with GCS score 13–15 and normal CT scan findings, 120 had positive MRI scans (43), while a systematic review with network metaanalysis revealed that diffusion MRI has higher sensitivity and accuracy compared to conventional CT and traditional MRI (44). Nevertheless, significant challenges remain for clinical use of diffusion MRI as a biomarker for TBI, since the FA or MD maps generated by DTI require statistical, not visual, interpretation (7).

The cluster analysis comparison found that some regions had significantly reduced FA values compared to healthy controls. The regions were superior fronto-occipital fasciculus, superior longitudinal fasciculus, uncinate fasciculus, and cingulum, as well as white matter fibers in the area of genu of corpus callosum, anterior corona radiata, superior corona radiata, anterior thalamic radiation and part of inferior frontal gyrus. Some major fiber tracts involved have been reported by previous TBI studies (2, 25, 41) as well as meta-analysis (9). Long tract fibers such as superior fronto-occipital fasciculus, superior longitudinal fasciculus and commissural fibers namely corpus callosum are particularly vulnerable to injury (45) and potentially causes disruption to information flow between brain areas that they interconnect.

These findings suggest that the white matter impairment may not be confined to a single region of the brain and could lead to stretching and distortion of axons as well as pathological changes in the myelin that evolves throughout the post-injury time course (46). Superior and inferior longitudinal fasciculus, and cingulum (14, 15), were areas reported to have significant FA value reduction that correlated with poor neuropsychological scores. Notably, the zones with altered FA seem to favor frontal regions; this could be possibly due to biomechanical factors tending to increase the mechanical distortion in the anterior part of the brain following TBI. The nature of the injury could play a role; a contre-coup injury in a motor vehicle accident often results in frontal lobe injury (47). Our sample of TBI patients, the majority of whom are motor vehicle accident patients, support this observation. The frontal and temporal lobes are areas known to be involved in memory including working memory (48), verbal memory and executive functioning (49), and the frontomedial circuits are important in the processing of episodic memory (50). There also seems to be a preponderance of right side FA reductions in our study. While white matter asymmetry has been shown to be a sequelae of traumatic brain injury (51), in our study, this may be attributed to the side of physical impact. However, the small sample size disallows further conclusion to be made as to whether it is due to an inherent aspect of TBI.

Our results further showed a significant reduction of Matrix Reasoning tests, and MAVLT Immediate that are consistent with findings of previous studies (26, 52). and both RCFT Immediate and Delayed Recall (53) scores in TBI patients compared to healthy controls.

The neuropsychological scores obtained in our study correlated with the reduced FA values in the areas where clusters were found in the TBSS analysis comparing TBI patients with controls. The MAVLT immediate recall was the most affected with positive correlation with the global FA difference between TBI and controls, and was found to specifically correlate with the FA in the genu of corpus callosum and right SFOF. Our results also showed positive correlation between matrix reasoning scores and FA values in the right SFOF and left ACR. Reviews and meta-analyses of the relationship between diffusion MRI findings and cognition found studies that support the positive relationship between FA values and cognitive scores especially in the domains of attention, memory and executive function (9, 54, 55). These meta-analyses also found that the strong relationship was not overly dependent on time post-injury. Reduced FA and increased MD in the subacute phase have been attributed to oedema and inflammation, while in the chronic phase, is suggestive of axonal injury followed by demyelination or gliosis (7).

On the other hand, our study found negative correlation between FA and matrix reasoning in the right SLF, while correlations performed using MD scores revealed positive correlation with RCFT immediate and delayed recall with the right SLF. A few previous studies also found instances of negative correlation, meaning having better cognitive score with reduced FA (9) in the acute stage (56) and years after injury (57). While the positive correlation between MD and RCFT scores, and the negative correlation between FA and matrix reasoning seem paradoxical, possible explanation may be that the relationship between FA and MD with neuropsychological scores are also affected by attentional and emotional factors as well as recruitment of neural resources in response to the task demand (58). Jantzen et al. (59) performed fMRI on post-concussion subjects and found higher BOLD activity in spatially larger brain region in a sequencing task compared to controls.

Previous studies have yielded mixed findings of the association between cognitive impairment and DTI parameters. We tested the TBI patients within 10 weeks post injury. Another study in TBI patients who had suffered severe and diffuse brain injury still performed worse than controls in the chronic (more than 2 years) stage of recovery (60). A meta-analysis by Roberts et al. (61) found a consistent relationship between DTI and cognitive functioning in pediatric patients more than 4 weeks but not in <4 weeks after TBI. A more recent study on geriatric age group patients showed that mild TBI with relatively high rates of neural degradation might put the patients at higher risk of developing Alzheimer's disease (AD), due to TBI modification of brain function along AD-like trajectories (62). Using machine learning, the acute assessment of cognitive performance has been shown to be able to prognosticate the occurrence of AD-like patterns of brain atrophy (22). A study by Wada et al. (25) found correlation between cognitive scores and FA values close to but not inside the reduced FA values in mild TBI patients. In response to the damages in certain areas of the brain, the other areas possibly undergo structural plasticity to compensate for the decreased function (63).

Recent development in this area have evidenced the possible existence of functional information in white matter (64), as shown by resting-state BOLD signals in white matter reflecting neural coding and information processing (65) as well as activation of white matter in direct response to perceptual and motor tasks (66–68). This functional connectivity of white matter is suggested to have a role in cognitive function (64).

A limitation of our study is the small sample size and male only gender which raises the possibility of bias. However, taken together with previous studies, it should be able to add to existing knowledge of subacute non-severe TBI in a different population. The limitation aspect of diffusion MRI should be considered. A false-negative result may occur because of crossing fibers or partial volume effect (69). We also used a diffusion MRI sequence with 32 diffusion directions. While a higher number of diffusion directions would be more sensitive in detecting white matter changes, the length of time it takes to scan in the patient group makes it less practical. In terms of management of TBI patients, although a group analysis approach may seem of little value in view of variabilities in anatomy, vulnerability to injury and injury mechanisms, standardization of acquisition and processing of MRI, as well as behavioral and cognitive assessments as biomarkers should be able to provide a clearer picture of TBI and its cognitive sequelae (70).

## Conclusion

In conclusion, the present study shows that patients with non-severe TBI have abnormally reduced FA values in multiple regions suggesting disruption of white matter tracts. The neuropsychological scores in these patients significantly correlated with the clusters of reduced FA values in specific areas.

## Data availability statement

The datasets presented in this article are not readily available because of ethical and privacy restrictions. Requests to access the datasets should be directed to JA, brainsciences@gmail.com.

## Ethics statement

The studies involving human participants were reviewed and approved by Human Research Ethics Committee of Universiti Sains Malaysia (USMJEPeM/15110486), Health Campus, Universiti Sains Malaysia, Kota Bharu, Kelantan, Malaysia. The patients/participants provided their written informed consent to participate in this study.

## Author contributions

Conceptualization and project administration: JA, AHA, and AIA. Formal analysis: ANA, AHA, and ST. Funding acquisition: JA. Methodology: JA, AHA, WW, and AIA. Supervision: AHA and RZ. Writing—original draft: ANA, AHA, and RZ. Data curation, investigation, resources, writing—review and editing, and approval of final manuscript: All authors.

## Funding

This work was funded by the Ministry of Higher Education Malaysia for Transdisciplinary Research Grant Scheme (TRGS) with project code TRGS/1/2015/USM/01/6/3, School of Medical Sciences through its Tabung Insentif Pembangunan Pengajian Siswazah PPSP (TIPPS), and USM fellowship scheme of Institute of Postgraduate Studies, Universiti Sains Malaysia.

## Conflict of interest

The authors declare that the research was conducted in the absence of any commercial or financial relationships that could be construed as a potential conflict of interest.

## Publisher's note

All claims expressed in this article are solely those of the authors and do not necessarily represent those of their affiliated organizations, or those of the publisher, the editors and the reviewers. Any product that may be evaluated in this article, or claim that may be made by its manufacturer, is not guaranteed or endorsed by the publisher.

## Abbreviations

TBI: Traumatic Brain Injury
TBSS: Tract-Based Spatial Statistics
CT: Computed Tomography
MRI: Magnetic Resonance Imaging
FA: Fractional Anisotropy
GCS: Glasgow Coma Scale
WASI: Wechsler Abbreviation Scale of Intelligence
MAVLT: Malay version of the Auditory Verbal Learning Test
WCST: Wisconsin Card Sorting Test
RCFT: Rey Complex Figure Test
CTMT: Comprehensive Trail Making Test
FSL: FMRIB's Software Library
FDT: FMRIB's Diffusion Toolbox
BET: Brain Extraction Tool
TFCE: Threshold-Free Cluster Enhancement
FEW: FamilyWise Error
AD: Alzheimer's Disease.
